# A Sham-Controlled Study of Neurofeedback for Pain Management

**DOI:** 10.3389/fnins.2021.591006

**Published:** 2021-07-26

**Authors:** Charlotte Ide-Walters, Trevor Thompson

**Affiliations:** ^1^Centre for Chronic Illness and Ageing, University of Greenwich, London, United Kingdom; ^2^Cancer Research UK, London, United Kingdom

**Keywords:** EEG-biofeedback, neurofeedback, experimental pain in humans, neuromodulation, sham-controlled design, acute pain, pain, sham-controlled

## Abstract

**Background:**

Neurofeedback (NFB) attempts to alter the brain’s electrophysiological activity and has shown potential as a pain management technique. Existing studies, however, often lack appropriate control groups or fail to assess whether electrophysiological activity has been successfully regulated. The current study is a randomized controlled trial comparing changes in brain activity and pain during NFB with those of a sham-control group.

**Methods:**

An experimental pain paradigm in healthy participants was used to provide optimal control of pain sensation. Twenty four healthy participants were blind randomized to receive either 10 × NFB (with real EEG feedback) or 10 × sham (with false EEG feedback) sessions during noxious cold stimulation. Prior to actual NFB training, training protocols were individually determined for each participant based on a comparison of an initial 32-channel qEEG assessment administered at both baseline and during an experimental pain task. Each individual protocol was based on the electrode site and frequency band that showed the greatest change in amplitude during pain, with alpha or theta up-regulation at various electrode sites (especially Pz) the most common protocols chosen. During the NFB sessions themselves, pain was assessed at multiple times during each session on a 0–10 rating scale, and ANOVA was used to examine changes in pain ratings and EEG amplitude both across and during sessions for both NFB and sham groups.

**Results:**

For pain, ANOVA trend analysis found a significant general linear decrease in pain across the 10 sessions (*p* = 0.015). However, no significant main or interaction effects of group were observed suggesting decreases in pain occurred independently of NFB. For EEG, there was a significant During Session X Group interaction (*p* = 0.004), which indicated that EEG amplitude at the training site was significantly closer to the target amplitude for the NFB compared to the sham group during painful stimulation, but this was only the case at the beginning of the cold task.

**Conclusion:**

While these results must be interpreted within the context of an experimental pain model, they underline the importance of including an appropriate comparison group to avoid attributing naturally occurring changes to therapeutic effects.

## Introduction

Chronic pain is one of the leading causes of disability (Van Hecke et al., 2013) and negatively impacts wellbeing, sleep, and physical health (Hadi et al., 2019; Murray et al., 2020), as well as costing billions in health care and lost work productivity (American Geriatrics Society Panel on the Pharmacological Management of Persistent Pain in Older Persons, 2009; Langley et al., 2010; Abdulla et al., 2013; Gaskin et al., 2017; Saxen and Rosenquist, 2020). Pain can also persist well after any physical injury has healed suggesting the central nervous system may play a significant role in the experience and maintenance of pain. Although the cortical signature of pain is complex, reliable evidence suggests involvement of a neuromatrix of cortical pathways (Melzack, 1999, 2001, 2005; Fitzgerald, 2020) including the anterior cingulate, prefrontal cortex, insular cortex, and primary and secondary cortices (Casey, 1980; Brooks and Tracey, 2005). These pathways may be mediated by several physiological (Apkarian et al., 2005) and psychological components (Wall et al., 1994; Thompson et al., 2012; Herbert et al., 2014; Riva et al., 2014; Dave et al., 2015). In theory, if the activity of the brain structures involved in pain processing can be regulated, this could in turn influence our experience of pain (Ros et al., 2010, 2013). This has led to an increased interest in novel interventions for pain management, such as neurofeedback (NFB).

NFB involves the real-time feedback of a person’s cortical activity by translating an EEG signal measured at the scalp to a changing audio or visual display (e.g., moving bars) in line with changes in the EEG. The basic aim of NFB is to provide a reward [e.g., auditory (music, chime), visual (point, token)] for a change in EEG activity that it is believed to be associated with a positive emotional or behavioral change, in line with basic principles of instrumental conditioning (Schabus et al., 2017). However, a recent review by Enriquez-Geppert et al. (2013) studied the role of NFB in the improvement of executive function (EF). Executive function is an important function that mediates learning, which highlights the link between both behavioral and cognitive elements involved in NFB training, and that there may be multiple factors that drive treatment efficacy (Enriquez-Geppert et al., 2013; Ros et al., 2020). There is a debate to the extent of which NFB can alter brain activity, and the exact mechanisms to how it operates (Gruzelier, 2014a,b; Ros et al., 2020), and reinforces the importance to conduct controlled studies in order to evaluate efficacy (Thibault et al., 2017).

Several studies have generated some promising findings supporting the potential of NFB for pain management (Roy et al., 2020). Some of the earliest studies were case studies (Sime, 2004; Kayiran et al., 2007). Sime (2004) studied a single female patient suffering from trigeminal neuralgia. It was concluded that the patient had experienced a large reduction in pain to the extent she canceled her planned neurosurgery, and even reduced her use of prescribed analgesic. Kayiran et al. (2007) conducted a case study of three patients who were diagnosed with Fibromyalgia (FMS) and also found pain decreased with NFB.

Later research examined larger cohorts of patients (Jensen et al., 2008, 2013a, 2014; Stokes and Lappin, 2010) and found promising findings. Jensen et al. (2007) conducted a study with 18 patients with complex regional pain syndrome and found a statistically significant decrease in pain reported pre to post training, with over half of participants reporting a clinically meaningful decrease of >30% (Moore et al., 2013). Stokes and Lappin (2010) and Jensen et al. (2013a, 2014) found similar results, however, Jensen et al. (2013a) found their decrease in pain was not clinically meaningful. In addition to single-arm designs, some studies have included control comparison groups (Kayiran et al., 2010; Caro and Winter, 2011; Hassan et al., 2015). Kayiran et al. (2010) implemented an active control group and Caro and Winter (2011) implemented an historic control group, and both found that their NFB groups demonstrated greater success than the control group for pain reduction. Hassan et al. (2015) attempted a placebo style control group, however, they did not have a dedicated control group and instead applied a sham style procedure to the 10th and 20th session for the same participants. The challenges of a cross over control design means that the participants will have possibly already learnt NFB and it isn’t an isolated controlled condition and the training they received previously may still influence how they respond during the non-trained session. However, Hassan et al. (2015) found promising findings with decreases in pain noted for the NFB sessions, and no reported decrease in pain for the sham conditions. All studies found results that offered support for the efficacy of NFB with EEG, however, it was recommended further controlled studies were required.

Importantly, the majority of these supporting studies lack an optimal placebo control groups and only a few studies have assessed whether cortical regulation (the key putative mechanism) has actually occurred Roy et al. (2020). Rogala et al. (2016) identified little evidence for desired changes in EEG frequency power. Some studies have found pain changed regardless of whether changes in EEG activity occurred (Jensen et al., 2013b). A sham-control group is the most appropriate research design to study a NFB intervention as it provides a method of controlling for several components that are fundamentally unrelated to EEG regulation but may nevertheless affect pain or EEG (Schabus et al., 2017; Roy et al., 2020), including attention and expectancy effects (Loo and Barkley, 2005, Thompson et al., 2011). If distraction, for example, is a key putative mechanism that underlies any analgesic effects of NFB treatment, it might be possible that such effects could be more simply and easily achieved using a simple distraction task (Thompson et al., 2011).

Most studies of NFB and pain have used protocols that focus on regulation of EEG activity at a target site expected to reflect cortical activity at key areas of the “pain matrix.” Imaging studies have established that the anterior cingulate, insular, primary/secondary cortices and the thalamus are active during naturally occurring and experimentally induced pain, with additional involvement of the prefrontal cortex in chronic pain reflecting it’s greater cognitive-emotional component (Apkarian et al., 2005). The variation of areas involved in pain processing is mimicked by considerable variation in the protocols used in previous studies. These have ranged from up-regulation of SMR at C4 (Kayiran et al., 2010) and theta at AFz (Jensen et al., 2018) to down-regulation of theta at Cz (Caro and Winter, 2011) or C2-C4 (Vučković et al., 2019), with details not reported in several other studies (Roy et al., 2020). A recent systematic review published in this Frontiers Research Topic (Roy et al., 2020) provides an extensive review of these protocols and concludes that no two protocols used across the examined studies were identical.

Most commonly, studies of NFB and pain have used a single fixed protocol applied to all participants. While such a generalized approach would be expected to be most beneficial for conditions with a predictable and homogenous pattern of EEG activity, a more effective approach where heterogeneity is present may be the use of individualized protocols based on the pattern of EEG dysregulation for that individual. Such data-driven protocols are typically based on an individual initial quantitative electroencephalogram (qEEG) assessment, which is compared to EEG from a normative database to identify the electrode positions and bandwidths to be targeted with the overarching aim of “normalizing” brain activity (Roy et al., 2020). This approach has recently been successfully adopted in NFB therapy for Chronic Tinnitus (Güntensperger et al., 2019) and ADHD (Dobrakowski and Łebecka, 2020). Despite the existence of an established cortical pain matrix, variation in EEG activity at different frequency bands and scalp sites is still likely as a result of heterogenous pain aetiology and individual factors such as head shape, and a few studies have utilized individualized protocols. Jensen et al. (2007), for example, used initial SMR up-regulation at T3/T4 in individuals with Complex Regional Pain Syndrome, but then employed progressively different protocols if the patient failed to report improvement until an optimal individual protocol was found. Prinsloo et al. (2018) used patient-specific protocols and found NFB to reduce pain in cancer survivors (although the authors did not report details of the electrode sites or frequencies that were trained).

In the current study, we will examine the effect of NFB within an experimentally induced cold pain paradigm applied to healthy participants. This approach has the advantage of facilitating the identification of an individualized protocol by comparing each participant’s EEG during a pain-free state with their EEG during noxious stimulation to identify the most relevant target site and frequency band. The use of experimentally induced pain also offers a level of control that can help overcome some of the difficulties encountered in clinical settings, such as day-to-day variation in chronic pain and use of pain medications that can complicate interpretation (Staahl et al., 2009). The two primary aims of the current study are to assess the ability of NFB to: (1) modulate cortical activity during pain; and (2) produce reductions in pain that exceed those of a sham-control group.

## Materials and Methods

### Participants

The sample consisted of 24 healthy volunteers with a mean age of 27.9 years (*SD* = 12.3, range = 18–56) with 9 males and 15 females. There was a mixture of 11 students (from the hosting University) and 13 non-student volunteers. Students were compensated with course credit for their participation, non-students were volunteers who received no payment or compensation for their time. Participants were required to confirm they did not violate any of the exclusion criteria, which were any long-term pain condition, Raynaud’s disease or any other condition that might affect the perception of or cause an adverse reaction to pain. Participants were also requested to abstain from the following prior to experimentation to prevent potential disruption of pain processing or EEG activity: analgesics (48 h); alcohol (12 h); caffeine (2 h); and nicotine (1 h). Participants were asked if they had ever taken part in a cold pain experiment or an experiment with EEG. All participants (*n* = 24) stated that they had not.

Participants were assigned to either the sham control group (*n* = 12) or the NFB group (*n* = 12) using block randomization to ensure equal group sizes. Participants were aware that they may be allocated to either the NFB or the placebo group but were blinded to group assignment until the end of the study. The researcher was not blinded as this was part of doctoral research and therefore it was the same researcher designing the study and implementing the research and analysis. Despite randomization of participants there was some difference between groups in the distribution of sex and age, with more females in the placebo group (*n* = 9) than the NFB group (*n* = 6), and with a lower mean age for the placebo (22 years) relative to the NFB group (31 years). As only healthy participants were used no “standard of care” equivalent was possible, however for all baselines the participants not in pain data was used as a baseline to both inform protocol selection and to set the target threshold for the NFB protocols.

### Sham Control Group

The sham-control group and the NFB intervention group underwent identical procedures, including assessment of individualized training sites for their NFB training (see section “Individualized Neurofeedback Protocols”), except for the feedback provided to them during the session. Specifically, the NFB intervention group were presented with genuine real time EEG activity and the sham-control group were given false EEG feedback using pre-recorded EEG data from participants who had undergone genuine NFB training.

### Pain Induction and Assessment

**FIGURE 1:**
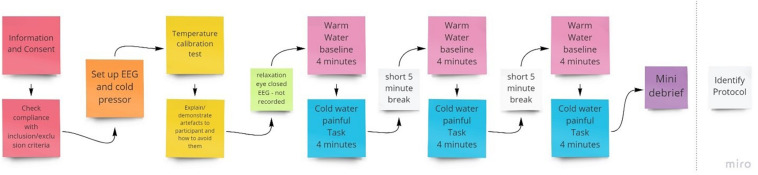
Procedure for the initial qEEG session to identify individual training protocols to be used in subsequent NFB sessions.

For pain induction, we employed the cold pressor task. A thermostatically controlled tank was used, which housed a circulating motor that ensured the water was consistently and continuously circulated around the tank to avoid localized heating of the hand. This method of pain induction is simple to administer with no long-term adverse effects (Mitchell et al., 2004). Further to this, the cold pressor test scores high in terms of validity and is widely used across several research areas to induce pain (Rebbeck et al., 2015). As shown in Figure 1, 3 × 4 min cold water pain inductions were administered within each session of NFB. Participants were told to take their hand out of the water at any point if they found the pain unbearable.

**FIGURE 2:**
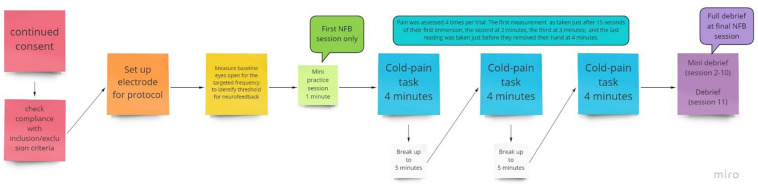
Flow chart of the procedure for an individual session, with NFB or sham given during the 3 pain tasks provided.

For pain assessment, a 0–10 numerical rating scale (NRS) was used to assess pain in response to cold stimulation and was administered at multiple time points during each NFB session. Specifically, participants were asked to verbally report pain ratings at 15 s (for a baseline measure) and at 2, 3, and 4 min during each of the three 4-min cold trials that took place during each session, for a total of 12 ratings per session (see Figure 2). The NRS is a well-established pain measure with a low administration time of just a few seconds, which was necessary to minimize disruption during the NFB session. For the purposes of analysis, NRS pain scores were averaged across the 3 trials that occurred with a session to produce a single pain rating at each of 15 s, 2, 3, and 4 min intervals.

### Individualized Neurofeedback Protocols

To inform electrode placement for the individualized sessions, a full brain EEG was first performed during a 4-min baseline warm (37°C) followed by a 4-min noxious cold (initially 10°C) trial, with this procedure conducted three times (Figure 1). The purpose of this was to identify optimal training electrode sites which best reflect changes in EEG during pain by comparing the individual’s EEG activity (amplitude) when in pain, subject to cold thermal noxious stimulation, and when not in pain (qEEG). For EEG recording, we used a Mitsar 202-32 EEG amplifier (REF), which has 31 active channels and 1 reference channel. Electrodes were placed according to the extended 10–20 system (channels included were: FPz, FP1, FP2, F7, F3, Fz, F4, F8, FT7, FC3, FCz, FC4, FT8, T3, C3, Cz, C4, T4, TP7, CP3, CPz, CP4, TP8, T5, P3, Pz, P4, T6, O1, Oz, O2; Thompson et al., 2008) and EEG recorded at 256 Hz, with all electrode impedances maintained at < 5 kΩ throughout. A common reference of the average signal across both ears was used during EEG acquisition.

After removal of EEG artifact, peak amplitude was computed for the painful cold and the non-painful warm condition for each electrode, for each of the three 8-min trials. The electrode site and frequency band for NFB training for a participant was selected based on the placement site for each individual and frequency that appeared to demonstrate the largest amplitude difference between the painful and non-painful stimuli, whilst also demonstrating consistency across the three trials. This is a similar method implemented by Stokes and Lappin (2010) who collected data from 10 sites to determine peak amplitudes and used this information along with prior experience to determine 5 homologous protocols to target their feedback. See Table 1 for the final protocols used.

**TABLE 1:** Protocols identified as optimal for the intervention and the (untrained) sham control groups.

Group	Frequency	Direction	Target site
NFB	Alpha	Up	Pz
NFB	Theta	Up	Fpz
NFB	Alpha	Up	C4
NFB	Alpha	Up	Pz
NFB	Theta	Down	Pz
NFB	Alpha	Up	FCz
NFB	Theta	Down	Cz
NFB	Alpha	Up	Pz
NFB	Theta	Up	FCz
NFB	Theta	Up	FCz
NFB	Theta	Up	Fz
NFB	Alpha	Down	C3
Control	Alpha	Down	CP3
Control	Alpha	Up	Pz
Control	Alpha	Up	Fp1
Control	Alpha	Up	P4
Control	Alpha	Up	Pz
Control	Alpha	Up	C3
Control	Alpha	Down	Cp3
Control	Theta	Down	F4
Control	Alpha	Up	Cp3
Control	Alpha	Down	C4
Control	Alpha	Up	T3
Control	Alpha	Down	FCz

### Cold Pressor Temperature Calibration

A single fixed noxious cold temperature can produce a wide variation in subjective pain intensity (Graven-Nielsen et al., 2001) and may produce no pain in some participants and pain that cannot be tolerated for more than a short period in others, we used an individualized temperature to provide a relatively homogenous level of baseline pain across participants. Specifically, we used a stimulus intensity that was tolerable for that individual for 4 min (the duration of the pain stimulus) and that resulted in a rating of 4–6 on a 0–10 pain rating scale as this roughly approximates a moderate and clinically meaningful level of pain (Boonstra et al., 2016). For the first trial, a 10°C temperature was initially used. If a pain rating <4 was given, the procedure was repeated with a 1°C reduction in temperature (i.e., made colder), to make the task more painful, and was continued until a 4–6 pain rating was reported. If the trial was terminated before the 4 min had elapsed or pain ratings were > 6, the temperature was increased by 1°C in a subsequent trial. The final individual temperature reached was applied for that participant in all the following NFB sessions. A comparison of NFB experimental and sham control groups with independent *t*-tests found no group differences in either temperature [*t* = 0.65, *p* = 0.52, *M* (NFB) = 10.2°C, *M* (Sham) = 9.8°C] or baseline pain intensity [*t* = 0.04, *p* = 0.97, *M* (NFB) = 4.52, *M* (Sham) = 4.54].

The entire first session took between 90 and 120 min to complete. This included time to brief and debrief, set up the equipment, and conduct assessments and the trials. Participants were also shown their EEG and the experimenter explained the implications of artifacts and showed how they are caused and how to avoid them to help reduce avoidable artifacts (Thompson et al., 2008).

### Neurofeedback

**FIGURE 3:**
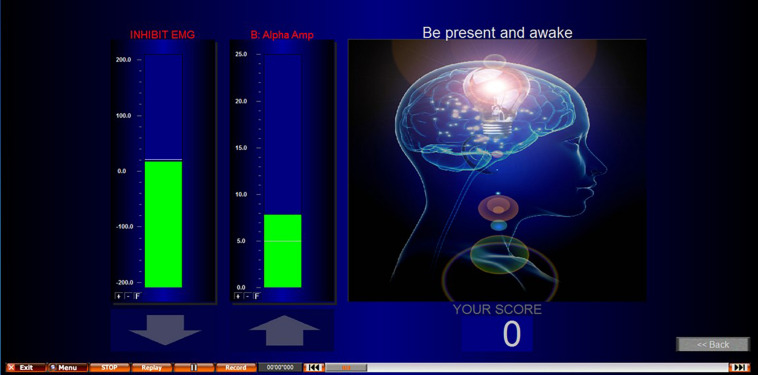
Screenshot of NFB screen presented during NFB/sham. EMG inhibit bar encouraged participants to keep their movement artifact low, and to prevent conditioning participants to achieve the desired outcome through movement. This second column shows an alpha uptraining example. However, alpha up, alpha down, theta up, and theta down were all protocols used in this study.

NFB was performed using a Nexus-4, which has up to 2 EEG channels recorded at a sampling rate of 256 Hz. Electrode placement was determined by an individual assessment of each participant’s EEG during pain as previously described (see section “Individualized Neurofeedback Protocols”). Biotrace software was used for the application of the NFB protocol and to build the biofeedback screen used (Figure 3 provides a screenshot). The reward protocol was to feedback a reward to the participant of 1 point for every second they were able to maintain their EEG within the targeted range.

The threshold (target) for the NFB protocol was calculated by recording 4 min of eyes open activity using the feedback electrode. The participants were not subject to any pain, but their hand was immersed into warm water as done above for the first baseline to identify the NFB protocol. This was to give a target to reinforce that was related to the subject non pain state on that day and at that time. The average EEG amplitude from the 4-min was used. This was repeated at the start of every NFB session to account for variations (e.g., different times of day) and the same threshold was used for all three trials within that session. This baseline was recorded with the participant’s non-dominant hand immersed into the warm water. This identified what their normal frequency for the targeted protocol frequency was for that electrode site when not in pain and was the benchmark for which they are attempting to train their cortical activity to return to during training. In addition to the training protocol, an EMG (muscle/movement artifact) control was added to the NFB screen to encourage participants to keep their EMG artifact low. The inhibit EMG bar was displayed to the left of the NFB protocol and showed green when the participant kept this at the correct level, and red when EMG increased. To further encourage participants to keep their EMG low, participants would only achieve points/rewards for achieving their protocol when their EMG was also at the correct level. This control helped to prevent moving, EMG artifact, and participants from training themselves to move to achieve the NFB protocol.

Participants took part in 10 NFB sessions, which were conducted 3–7 times per week subject to participants’ availability, with no more than one session per day to avoid fatigue but to ensure it was regular. Each session lasted around 45–60 min, which included set up time, briefing/debriefing, baseline measurement and three cold trials (Figure 2). Each session consisted of 3 × 4 min cold pain tasks at the participant’s individualized temperature.

In the first of the ten NFB sessions, participants were given a minute to practice and understand the objective of the session. Participants were not provided with a strategy, only to be observant of when their cortical activity fed back that they had been successful and to try to replicate that. Their understanding was checked, and they were reminded about artifacts and how to avoid them. In each of the 4-min cold trials, pain ratings were recorded at 15 s, 2, 3, and 4 min or when they removed their hand. A break of up to 5 min was added in between each cold pain task in order to allow participants to recover from the numbing effects of the cold water.

### EEG Data Pre-processing

The data was visually inspected and if any large EMG artifacts were found these were removed manually before running independent component analysis (ICA) to identify EOG artifact. ICA was conducted using the WinEEG software with eye blink or lateral eye movement components removed automatically (Gao et al., 2010).

In the initial assessment of data for determining protocols, WinEEG was used to conduct Fast Fourier Transform (FFT) for Delta (0–4 Hz), Theta (4–8 Hz), Alpha (8–12 Hz), and Beta (12–40 Hz), visually comparing all spectral output (with a similar approach to Stokes and Lappin, 2010), by subtracting the baseline from the painful condition. For the main analysis, amplitudes for the target frequency bands (alpha or theta) were computed using FFT from the first 30 s and the last 30 s for each of the three trials within a session. These were then averaged across the three trials, at each time point within a session, to give a single mean amplitude for the first 30 s and the last 30 s of each of the 10 sessions.

### Statistical Analysis Plan

#### The Effect of Neurofeedback on Pain and Regulation of EEG

Mixed ANOVAs were used to examine whether changes in EEG occurred *during* sessions (first 30 s vs. last 30 s) and *across* sessions (1–10), and whether this differed by group (NFB vs. control). Given that the target EEG amplitude (established from the individual’s EEG activity during their pain-free baseline state) required up-regulation for some participants and down-regulation for others, change was coded such that a positive sign indicated a change in the desired direction (i.e., toward the target amplitude) while a negative sign indicated a change in the opposite direction. Although there are different ways in which training “success” could be potentially assessed, we computed the size of the difference between the participant’s target amplitude and the amplitude during the pain task (at the chosen training site and frequency band). Smaller differences therefore represent greater “success,” as reward protocols were designed to return amplitude to the target frequency associated with a baseline pain-free state.

To assess whether NFB resulted in lower pain than the control procedure during sessions (baseline 15 s, 2, 3, and 4 min) or across sessions (1–10), the same analysis was conducted as described above, but with pain ratings as the outcome variable. In all instances, trend analysis was also conducted to examine whether any progressive changes in EEG or pain across sessions followed a linear or quadratic trend.

## Results

### Data Screening

EEG data were missing for 5 of the 240 (2%) sessions due to equipment failure, and these values were imputed using the mean value from non-missing participants for the equivalent trial and session number. For pain rating data, < 2% of data were missing so we imputed missing values with the participant’s trial average. No outliers were identified (*z* > 3.29 or < −3.29, Tabachnick and Fidell, 2013) for pain ratings. Z scores for EEG amplitudes suggested 5 outliers which were again substituted with linear interpolations of that participant’s adjacent sessions. Alpha (*SD* = 2.9) and theta (*SD* = 2.1) frequencies showed similar dispersion.

### Regulation of EEG

Individual protocols for each participant in the NFB groups are shown in Table 1 (which also shows the protocols identified to be optimal for the sham-control participants although no actual NFB was performed). Training protocols for the NFB group consisted of a mixture of alpha and theta training (at different electrode sites), with six participants receiving alpha training (down-regulation = 1, up-regulation = 5) and six receiving theta regulation (down-regulation = 2, up-regulation = 4).

**FIGURE 4:**
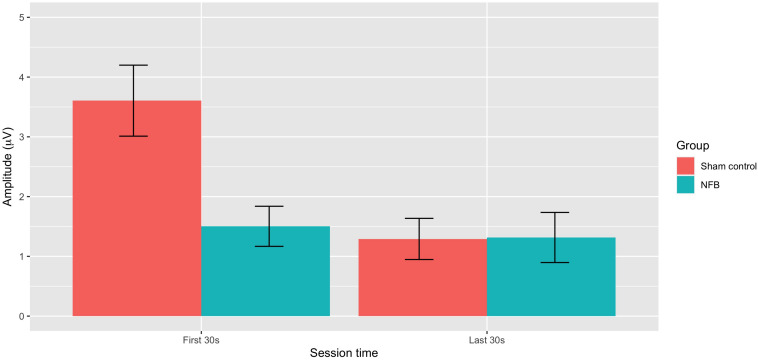
Mean absolute deviation from baseline EEG amplitude for NFB and sham control groups during the first 30 and last 30 s of the pain tasks (error bars represent ± 1 SE).

ANOVA revealed a significant overall deviation from target EEG *during* a session [*F*(1, 22) = 10.35, *p* = 0.004], with this deviation greater during the first 30 s (*M* = 3.01) compared to the last 30 s (*M* = 2.08) of the pain task. There was also a significant During-session X Group interaction [*F*(1, 22) = 10.31, *p* = 0.004], with Figure 4 suggesting EEG amplitude was closer to the target value during NFB than the control procedure during the first 30 s of the pain task, but with little difference near the end of the task. No other effects approached significance (*p* = 0.13–0.46), with all results shown in Table 2.

**TABLE 2:** Results of the Mixed ANOVA for EEG amplitude with *η^2^ indicating the partial-eta squared* effect size.

	*df*	F	η^2^	*p*-value
Group	1, 22	2.39	0.098	0.137
Across^a^ sessions	5.8, 127.9	0.96	0.042	0.455
During^b^ session	1, 22	10.35	0.320	0.004*
Group × Across sessions	5.8, 127.9	1.67	0.071	0.135
Group × During session	1, 22	10.31	0.319	0.004*
Across sessions × During session	6.2, 136.9	0.98	0.043	0.441
Group × Across sessions × During session	6.2, 136.9	1.38	0.059	0.226

*^*a*^Across sessions (1–10).*

^*b*^During a session (first 30 vs. last 30 s). *Significant <0.05.

As different protocols were used, we conducted an exploratory investigation of whether alpha training or theta training produced a greater impact on EEG parameters by rerunning ANOVA on the NFB experimental group only (a protocol was not implemented for the sham group), but including frequency band (alpha vs. theta) as an additional variable. Results found no significant differences between alpha (*M* = 2.06 μV) and theta (*M* = 2.24 μV) frequency bands (*p* = 0.798) or any interactive effects (*p* = 0.342–0.813). We did not compare up-regulation (*M* = 2.35 μV) vs. down-regulation (*M* = 1.55 μV) with inferential statistical tests given the fact only three participants were given down-regulation training.

### Pain Modulation

**FIGURE 5:**
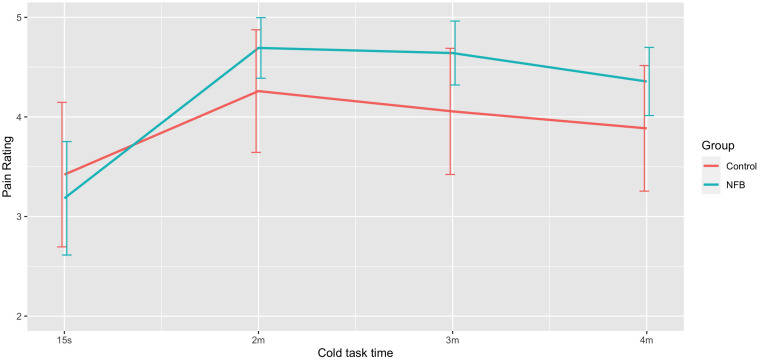
Mean pain rating at different stages of the 4-min pain trial for NFB and sham control groups (error bars represent ± 1 SE).

ANOVA revealed a significant difference in mean pain ratings *during* the session *F*(3, 66) = 7.34, *p* < 0.001), with Figure 5 showing a sharp rise in pain from 15 s to that at 2, 3, and 4 min period, with a quadratic trend identified [*F*(1, 22) = 24.31, *p* < 0.001]. Neither the control nor the experimental groups saw a clinically meaningful reduction in average pain ratings (>30%).

**FIGURE 6:**
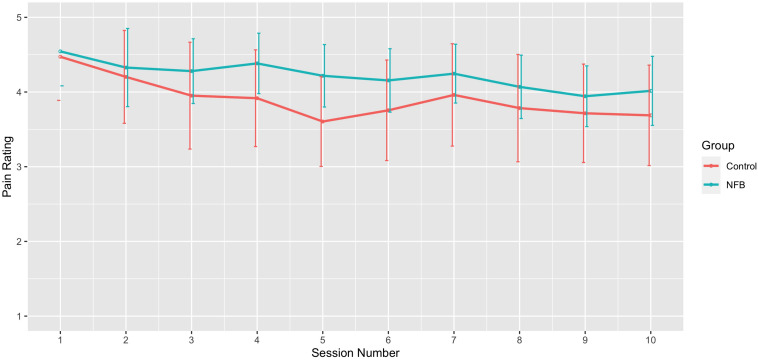
Mean pain rating across sessions for NFB and sham control groups (error bars represent ± 1 SE).

With respect to changes in overall pain *across* sessions, significant differences across the 10 sessions were also observed [*F*(9, 198) = 3.09, *p* = 0.002], with trend analysis indicating a progressive linear decrease in pain overall across sessions [*F*(1, 22) = 6.91, *p* = 0.015]. There was no significant interaction of during session changes and group, with both NFB and controls demonstrating a similar decrease in pain ratings across sessions as shown in Figure 6. Table 3 shows the complete table of results and with no other effects significant.

**TABLE 3:** Results of the Mixed ANOVA for pain ratings with η^2^*indicating the partial-eta squared* effect size.

	*df*	F	η^2^	*p*-value
Group	1, 22	0.23	0.010	0.637
Across^a^ sessions	9, 198	3.09	0.123	0.002*
During^b^ session	3, 66	7.34	0.250	< 0.001*
Group × Across sessions	9, 198	0.43	0.019	0.919
Group × During session	3, 66	0.92	0.040	0.437
Across sessions × During session	27, 594	01.45	0.062	0.069
Group × Across sessions × During session	27, 594	0.73	0.032	0.838

*^*a*^Across sessions (1–10).*

^*b*^During a session (baseline 15 s, 2, 3, and 4 min). *Significant <0.05.

We also conducted an exploratory investigation of whether pain ratings were impacted by the type of frequency band trained (alpha vs. theta) as described in the previous section. Results indicated no main (*p* = 0.853) or interactive effect of frequency band on pain ratings (*p* = 0.315–0.699).

## Discussion

Several key results emerged from the present study. First, although we individualized neurofeedback protocols based on each participant’s EEG response to painful stimulation, a general pattern of altered EEG activity in certain regions of the scalp at specific frequency bands emerged. In particular, alterations in alpha and theta frequency bands in mostly frontal, and some central, regions were observed, suggesting these may be key target areas for NFB for pain management. This is largely consistent with cortical areas found to be active during pain in imaging studies (Apkarian et al., 2005, 2009; Fitzgerald, 2020), but also supports the complex nature of pain and the need to better understand the relationship between the brain and pain on a cortical level (EEG) to develop efficacious NFB protocols with EEG.

Second, in terms of the ability of participants to regulate EEG activity when in pain, results indicated EEG amplitude at the training site during pain was closer to the target amplitude for the NFB than the sham group in the early stages of the pain task. Importantly, however, this was only observed during the first 30 s of the training task, with no differences between NFB and sham in the latter stages of the task (final 30 s). This pattern of results does not provide a clear-cut picture of whether EEG regulation during pain is possible. One possible interpretation is that EEG can be more successfully regulated (compared to the natural EEG occurs during pain) during the early stages of pain, but that this becomes more difficult after sustained exposure to noxious stimulation. However, it seems unlikely that any substantive training effects would be present so early on in the task (30 s), and any differences at this stage of the task seem more likely to simply reflect group differences in reaction to an initial noxious stimulus. This ambiguous pattern of findings does little to resolve the question of whether EEG can be regulated during pain, but does underline the complex nature of EEG changes that occur during pain and clearly indicates that more convincing evidence is required before this claims that EEG can be regulated during pain can be made.

These findings are consistent with some previous work. Hassan et al. (2015) reported that in control sessions of NFB, changes in power spectral density (PSD) did not differ across the placebo controlled condition to before the session (pre-NFB vs. during NFB), suggesting that training did not take place in the controlled condition; however, there doesn’t appear to be a direct comparison between groups to test for statistical significance. Kayiran et al. (2010) identified no significant changes in EEG amplitude overall, however, they identified a statistically significant difference for session 4. This suggests that further research is needed to better understand this, for example Aliño et al. (2016) and Roy et al. (2020) suggest additional variations of sham-procedures that could be run in parallel with an intervention to further study changes between intervention and sham groups, as well as double blinding (Ros et al., 2020), which future research could attempt with pain. Overall, these results are consistent with the possibility that NFB can elicit changes in EEG during pain that are different from those that occur with a sham procedure, although the fact that this seemed to occur only in the early stages of noxious stimulation does underline the need for further corroborating evidence.

With respect to the potential for NFB as a technique for pain management, we found no differences in pain ratings between NFB and sham control either during the sessions or across sessions. We did, however, find a decrease in average pain intensity with progressive sessions that were very similar for both the NFB and sham control groups. This could provocatively suggest that any putatively beneficial effects on pain before and after a series of NFB session observed in previous studies that do not provide a sham comparator may have been attributable simply to non-EEG regulatory mechanisms such as placebo expectation effects, demand characteristics or a number of other potential explanations (Loo and Barkley, 2005; Thompson et al., 2011; Gollub et al., 2018).

Although our study involved experimentally induced pain in healthy participants, rather than clinical pain, these findings do nevertheless demonstrate that changes in reported pain can occur in the absence of real NFB and this may be critical to the interpretation of any possible therapeutic effects. There appears to be little previous research that has compared changes in pain during NFB to a sham procedure, and the current findings endorse the view of Aliño et al. (2016) that a suitable sham procedure is essential to identify any clear benefits of NFB. One study by Hassan et al. (2015) has attempted to test for a placebo effect in two ways. One involved displaying a pre-recorded NFB session and the other involved displaying visual feedback and targeting Oz (the occipital area), which is not normally associated with pain. This was done on the 10th and 20th training session for participants so there was no independent sham-control group. As mentioned previously this limitation means that learning or other carryover effects cannot be ruled out. Hassan et al. (2015) found that the targeted activity for participants did not change in the placebo-controlled group studying PSD, nor was a reduction in pain reported. The alpha placebo control did note a shift in alpha, but again no change in pain reported. Although these findings seem promising, this design does not allow conclusions of the efficacy of NFB to be made and further controlled studies were required. This study has attempted to address this and is able to make direct comparisons between a sham-control group and a NFB intervention group, including a manipulation check to not only study if EEG changed, but if it was changing as was intended/trained.

The current study has several limitations. First, it is impossible to say from our results whether NFB *per se* is ineffective for reducing pain, or simply that there is limited evidence for the effectiveness of the NFB approach we examined. Given the almost endless variation in how neurofeedback can be administered (e.g., a differing number and length of sessions, single or multiple channel approaches, outcomes targeted, different reward protocols etc.), it would be unwise to dismiss the possibility of therapeutic effects of NFB. Given encouraging findings reported elsewhere, further work is clearly required to identify which of these factors might be important in potentially affecting the success of NFB. Second, while the use of an experimental pain paradigm offers a high degree of control, results do not necessarily generalize to clinical pain which differs on intensity, perceived control and frontal cortex involvement (Apkarian et al., 2005). Despite this, there are many cortical areas that are commonly active during the processing of both chronic and acute pain and therefore does have the potential to be effective, these findings suggest that the most likely target is the frontal and central areas area (e.g., FCz, Pz). Third, if a genuine analgesic effect of NFB does exist, it may simply have been that the current study was underpowered to detect this. As there are few if any studies that have examined the impact of NFB on experimentally induced pain we were not able to estimate an effect size that could be used *a priori* to perform power analysis calculations. Although the *p*-value for pain indicated a reliable decrease in pain ratings across sessions (*p* = 0.015) and the present sample size is similar to others in the field, a larger sample size in future could provide greater power for group comparisons. Fourth, despite random group allocation there were some chance differences in age and gender across groups, with the NFB group 9 years older on average with more females compared to the sham group (9 vs. 6). As we examined NFB effects within a repeated-measures design, we would not expect these differences to have had any substantive impact on an evaluation of therapeutic effects, in the absence of any previous robust evidence of any major moderating effects of these variables. Nevertheless, stratification may be a sensible option in future studies to avoid this possibility given it is often difficult to recruit large numbers of people in NFB studies needed for randomization to be most effective.

Overall, these findings underline the importance of a robust study design which includes an appropriate sham control group in order to reliably evaluate the possible therapeutic effects of NFB. In particular, the fact that an analysis of the NFB group in isolation would have indicated a successful reduction in pain across progressive sessions and thus a treatment benefit, despite a similar benefit being observed in the sham group, does prompt extreme caution in interpreting previous studies indicating NFB benefits where no sham control group is present. Further studies including those with clinical pain populations that include an appropriately designed sham control group are needed to clarify the therapeutic potential for NFB as a pain intervention.

## Data Availability Statement

The raw data supporting the conclusions of this article will be made available by the authors, without undue reservation.

## Ethics Statement

The studies involving human participants were reviewed and approved by Secretary, University Research Ethics Committee c/o Vice Chancellor’s Office, Queen Anne Court, University of Greenwich, Old Royal Naval College, London. The patients/participants provided their written informed consent to participate in this study.

## Author Contributions

TT and CI-W designed the study. CI-W recruited participants, collected and analyzed the data, and wrote the initial manuscript. TT conceived the study, contributed to the writing of the manuscript, provided statistical advice, and provided a critical review of the final draft. Both authors contributed to the article and approved the submitted version.

## Conflict of Interest

The authors declare that the research was conducted in the absence of any commercial or financial relationships that could be construed as a potential conflict of interest.

## Publisher’s Note

All claims expressed in this article are solely those of the authors and do not necessarily represent those of their affiliated organizations, or those of the publisher, the editors and the reviewers. Any product that may be evaluated in this article, or claim that may be made by its manufacturer, is not guaranteed or endorsed by the publisher.
